# Massive Device-Related Thrombus After LAA Occlusion: Intraoperative Insights Into Mechanism

**DOI:** 10.1016/j.jaccas.2022.07.037

**Published:** 2022-11-02

**Authors:** Damir Vukomanovic, Samuel Unzek, Kashif Malik, Alicia Taase, Michael Zawaneh, Peter Weiss, Kenith Fang, Roderick Tung

**Affiliations:** a Department of Internal Medicine, University of Arizona College of Medicine, Banner University Medical Center-Phoenix, Phoenix, Arizona, USA; b Division of Cardiology, University of Arizona College of Medicine, Banner University Medical Center-Phoenix, Phoenix, Arizona, USA; c Department of General Surgery, University of Arizona College of Medicine, Banner University Medical Center-Phoenix, Phoenix, Arizona, USA; d Department of Cardiothoracic Surgery, Banner University Medical Center-Phoenix, Phoenix, Arizona, USA

**Keywords:** atrial fibrillation, device-related thrombus, left atrial appendage occlusion device, Watchman, AF, atrial fibrillation, DRT, device-related thrombus, INR, international normalized ratio, LAA, left atrial appendage, LAAO, left atrial appendage occlusion, TEE, transesophageal echocardiogram

## Abstract

In patients with a contraindication to oral anticoagulation, the left atrial appendage occlusion devices are an approved alternative. Device-related thrombus is a recognized complication, but underlying mechanisms are incompletely understood. In this case series, the authors describe potentially the same mechanism of thrombosis with intraoperative images of incomplete endothelialization. (**Level of Difficulty: Intermediate.**)

## Central Illustration

**Figure:**
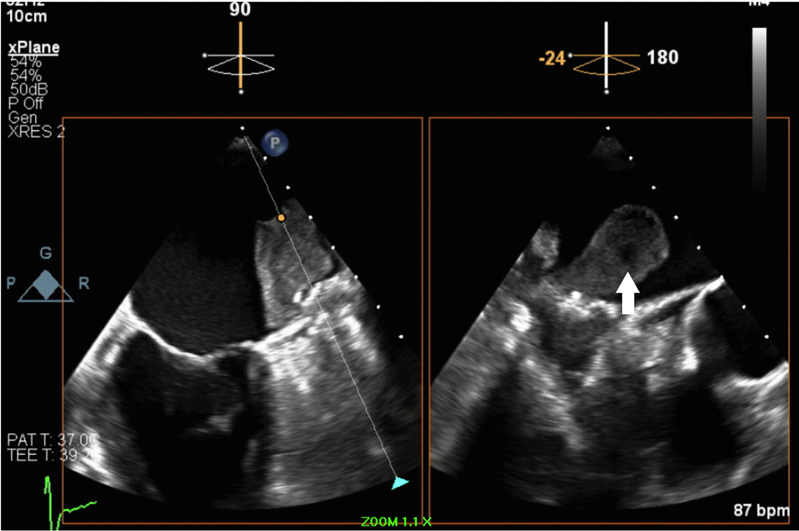

Patients with atrial fibrillation (AF) who require long-term anticoagulation for stroke prevention with a contraindication to oral anticoagulation may benefit from left atrial appendage occlusion (LAAO). The Watchman device (Boston Scientific) has been most extensively studied; however, complications like device-related thrombus (DRT) formation may occur after device implantation. In the largest prospective study consisting of 1,739 participants, the incidence of DRT across both the PROTECT-AF (Watchman Left Atrial Appendage System for Embolic Protection in Patients With Atrial Fibrillation) and PREVAIL (Evaluation of the Watchman Left Atrial Appendage Closure Device in Patients With Atrial Fibrillation Versus Long Term Warfarin Therapy) trials, as well as 2 prospective registries (CAP2 [Continued Access to PROTECT-AF] and CAP [Continued Access to PREVAIL]), was approximately 3.74%.^1^ The mechanism underlying DRT is incompletely understood. Here, we describe a series of 2 massive DRT cases with novel intraoperative images of the device after thrombus resection, and review the limited literature that exists regarding its management.

**Learning Objectives**

• To understand the potential late complication and mechanism of thrombus formation after left atrial appendage occlusion device implantation.
• To understand the management options for this complication and identify knowledge gaps in this subject.

## Case 1

A 75-year-old woman with a history of hypertension, diabetes mellitus type 2, and permanent AF with a CHA_2_DS_2_-VASc (congestive heart failure, hypertension, age ≥75 [doubled], diabetes, stroke [doubled], vascular disease, age 65 to 74 and sex category [female]) score of 5 and HAS-BLED score of 4 underwent LAAO (Watchman, 27 mm; Boston Scientific). She was discharged on warfarin and aspirin 81 mg for 45 days. A transesophageal echocardiogram (TEE) 2 months after implantation revealed no thrombus or peridevice leak, so she was transitioned to aspirin and clopidogrel.

At the scheduled 1-year postimplantation TEE, a large 2.0 × 2.0-cm nonmobile thrombus adherent to the LAAO device was detected. The patient was restarted on warfarin, and after 6 months, the TEE revealed persistent thrombus of the same dimensions despite a consistently therapeutic international normalized ratio (INR). Warfarin was discontinued, and dabigatran 150 mg twice a day was initiated, diminishing the thrombus size significantly by 3 months. Unfortunately, the patient had a severe gastrointestinal bleed requiring blood transfusions, and dabigatran was discontinued. The patient was maintained on monotherapy with aspirin 81 mg daily.

**Figure 1:**
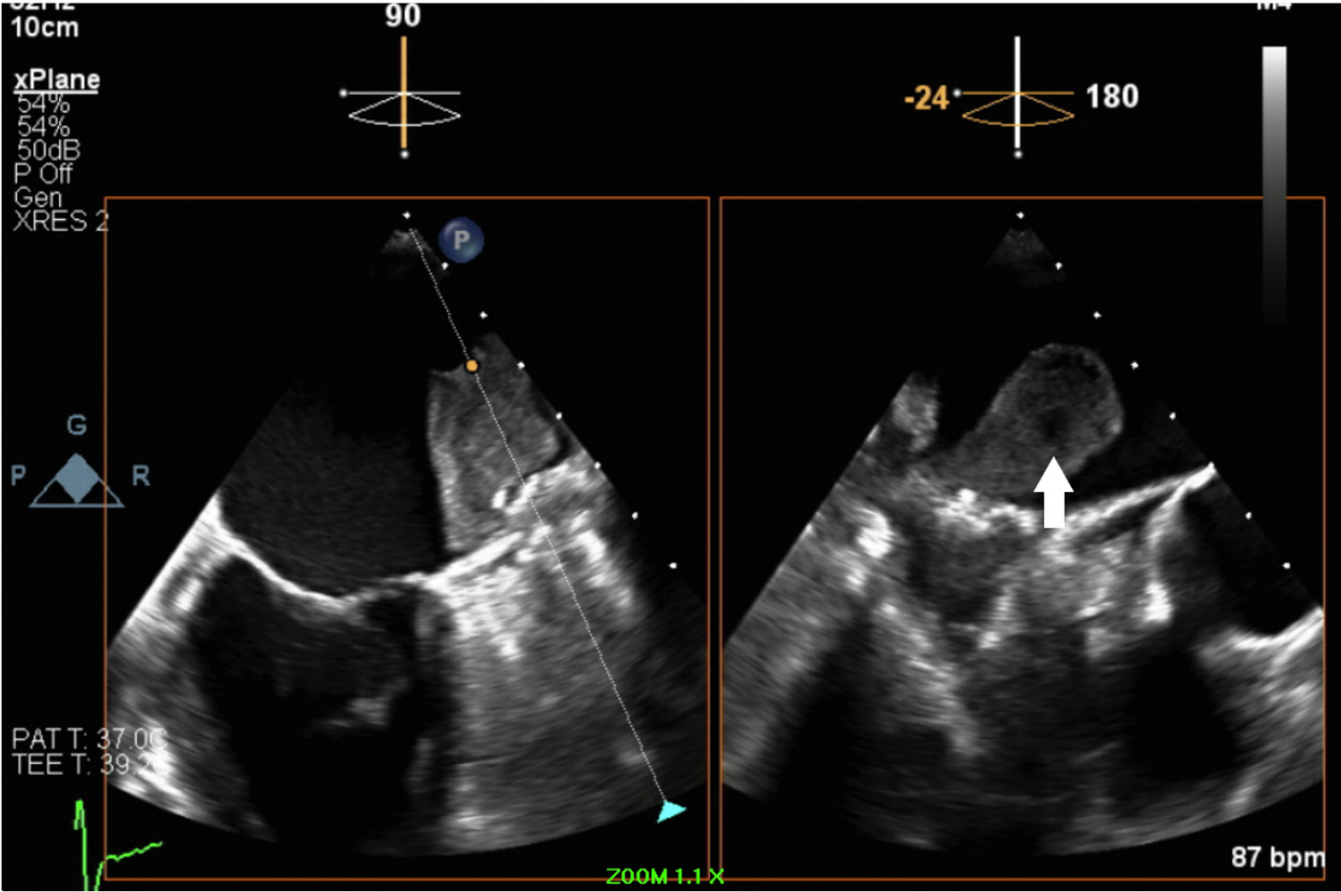
Transesophageal Echocardiogram Findings: Case 1 Biplane transesophageal echocardiogram still frame image featuring a 3.5 × 2.2-cm thrombus situated on the left atrial appendage occlusion device with various degrees of maturation **(white arrow)**.

**Figure 2:**
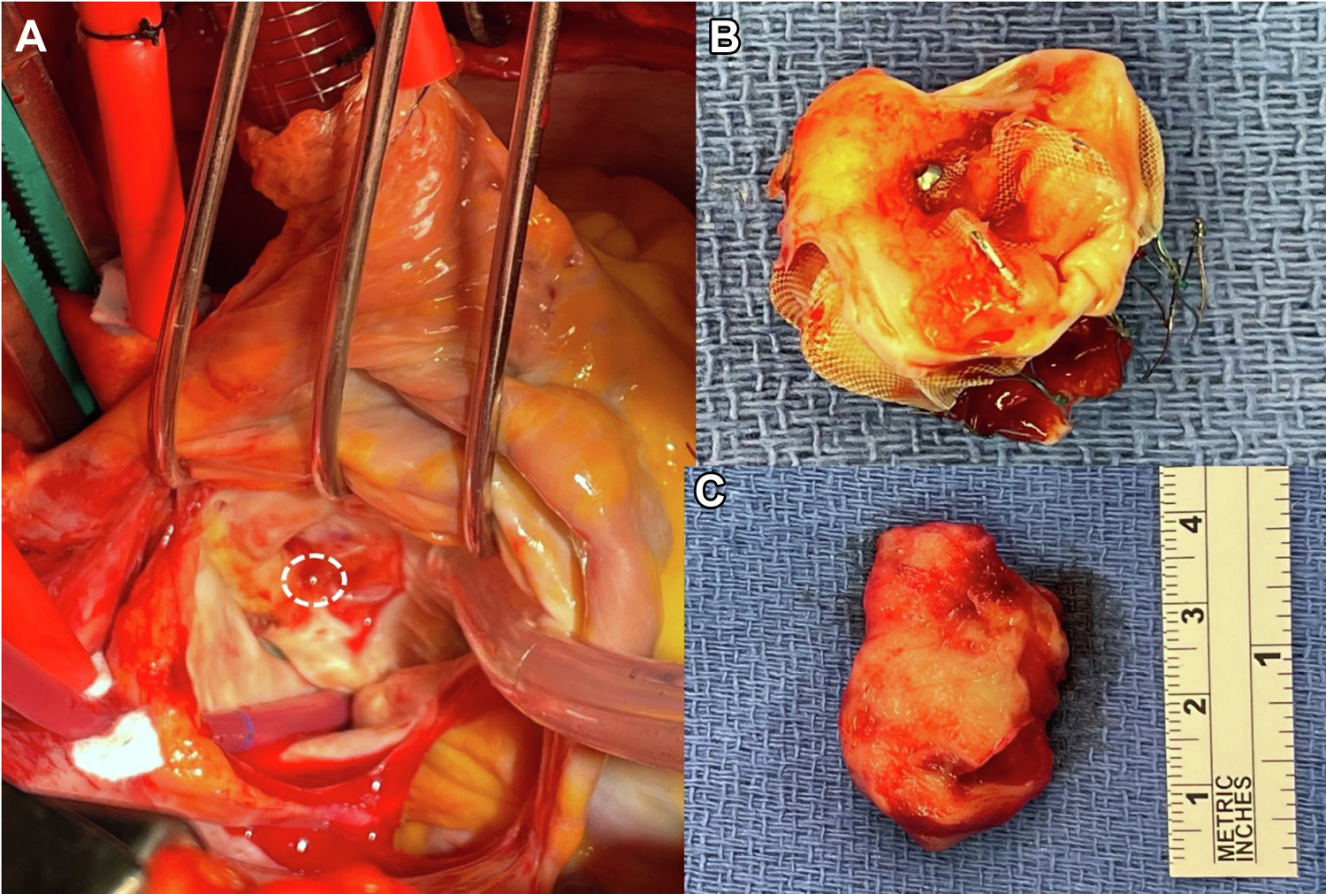
Intraoperative Findings: Case 1 **(A)** Nonendothelialized central screw hub in **dashed circle**. **(B)** Excised left atrial appendage occlusion device encased in organized thrombus. **(C)** Organized thrombus excised from above the device.

One year after dabigatran was discontinued (3 years postimplantation), a routine TEE showed a massive mobile 3.5 × 2.2-cm thrombus attached to the LAAO device (Figure 1). The patient underwent successful left atrial thrombus extraction with LAAO device retrieval and complete surgical resection of the LAA. Careful intraoperative examination of the LAAO device revealed a nonendothelialized central screw hub, which was present 3 years after original implantation (Figure 2). She was recently seen in clinic and doing well.

## Case 2

**Figure 3:**
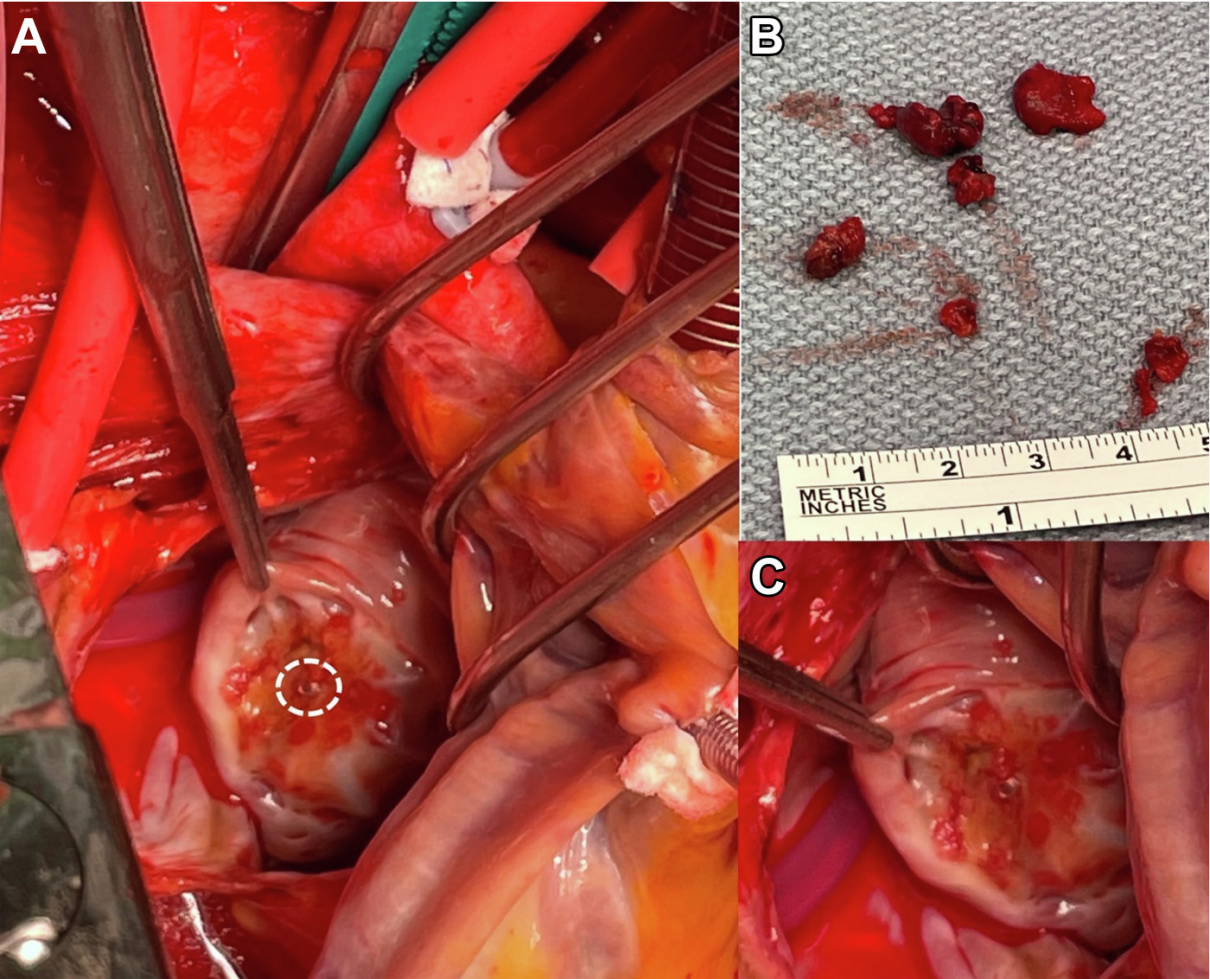
Intraoperative Findings: Case 2 **(A)** Nonendothelialized central screw hub in **dashed circle**. **(B)** Excised organized thrombus removed in fragments from the left atrial appendage occlusion device. **(C)** Additional view of incompletely endothelialized central screw hub.

A 75-year-old woman with a past medical history of hypertension, hyperlipidemia, moderate mitral and aortic valve regurgitation, and permanent AF not on anticoagulation because of frequent gastrointestinal bleeding underwent LAAO (Watchman, 31 mm; Boston Scientific). Her CHA_2_DS_2_-VASc score was 3 and HAS-BLED score of 3 at that time. There was no evidence of thrombus or leak on TEE performed 45 days post-deployment. After 6 months of anticoagulation and dual antiplatelet therapy, she was placed on aspirin 81 mg daily. Months later she began having dyspnea on exertion that progressed. During her last echocardiographic evaluation, she was found to have progression of her valve disease. Twenty-six months after LAAO, she underwent an aortic valve replacement, a mitral valve repair, and COX-maze. Intraoperatively, the LAAO device was well-seated in the LAA, but during close inspection, a 2.5 × 2.5-cm thrombus was identified sitting on the base surface in the cephalad position of the LAAO device. The thrombus was removed in fragments and, similar to case 1, the central screw hub was nonendothelialized and exposed on direct examination (Figure 3). She was last seen 2 months ago without stroke.

## Discussion

We report novel intraoperative findings in 2 patients presenting with DRT associated with an LAAO device despite routine dual antiplatelet therapy. Intraoperative examination revealed incomplete endothelialization of the central screw hub in both cases. These provide a potential mechanistic insight into the development of DRT associated with the LAAO device. To our knowledge, only 1 prior intraoperative image with a persistently exposed central screw has been published by Ellis and Piccini^2^ in an accompanying editorial to Dukkipati et al.^1^

Described risk factors for DRT include device size, preexisting LAA thrombus, female sex, smoking, subtherapeutic INR/oral anticoagulant noncompliance, and a high CHA_2_DS_2_-VASc score often ≥3.^3^^,^^4^ The exposed and incompletely endothelialized screw observed in these cases cannot be definitively concluded to be the sole mechanism of DRT. Important factors that may also contribute to increased thrombogenicity include stasis from left atrial dilation, diminished left ventricular function, hypercoagulability from system illness, implantation depth, and genetic resistance to antiplatelet therapy.^1^^,^^5^^,^^6^ However, both cases presented had normal left ventricular ejection fraction, no evidence of spontaneous echo contrast, with implant depths <10 mm (1 mm, 2 mm).

With limited data available on endothelialization of the LAAO device, a preclinical study done in canines reported histopathologic endothelialization of the LAAO device at 45 days, and thus a potential basis for the duration of initial anticoagulation.^7^ Given likely heterogeneous patient-specific endothelialization processes, extending the window of antiplatelet and anticoagulation may decrease the incidence of DRT. Newer device designs with lesser extent or shorter exposed central screws, like the Watchman FLX, may lower the risk for DRT.

In the event of a DRT, there is no clear guidance on optimal treatment strategies. A systematic review by Lempereur et al^8^ examined the treatment of the LAAO device and atrial septal defect occlusion DRT, finding that low molecular weight heparin achieved 100% thrombus resolution and oral anticoagulation achieved 89.5% thrombus resolution at 14 days and 90 days of treatment, respectively. If DRT occurred while on warfarin, some studies increased the INR goal to 2.5 to 3.5 for 8 to 12 weeks, or switched to direct oral anticoagulants, which have limited outcome data.^8^

In patients with DRT who are at high risk for bleeding or have other contraindications to anticoagulation, surgical options are available. The specific size of thrombus that warrants surgery remains unknown, and here we report thrombi larger than 1 cm. Akin to endocarditis management, there may be a size cutoff (>1 cm) that warrants surgical management.^9^ Although data remain limited and currently based mainly on case reports, outcomes appear to be satisfactory. Interventions such as LAA ligation after LAAO removal to autologous pericardial patch over the LAAO device to prevent further thrombus formation have been tried.

## Take-Home Points

We describe 2 cases of massive DRT that formed despite completion of standard anticoagulation and without resolution despite continued alternative anticoagulation management. As many submassive cases do not necessitate surgical intervention, intraoperative findings are rare.

• Gross examination of the retrieved devices showed incomplete endothelialization with persistently exposed central screws at 3 and 2 years postimplantation, respectively.
• Prompt anticoagulation appears to be an effective option for most DRTs, with monitoring for thrombus regression or progression.
• In the event of massive thrombus or contraindication to anticoagulation, surgical intervention can result in safe resolution of massive DRT.

## Funding Support and Author Disclosures

The authors have reported that they have no relationships relevant to the contents of this paper to disclose.
